# Should we encourage the use of robotic technologies in complicated diverticulitis? Results of systematic review and meta-analysis

**DOI:** 10.3389/frobt.2023.1208611

**Published:** 2023-09-13

**Authors:** S. I. Panin, T. V. Nechay, A. V. Sazhin, A. E. Tyagunov, N. A. Shcherbakov, A. V. Bykov, K. Yu Melnikov-Makarchuk, A. G. Yuldashev, A. A. Kuznetsov

**Affiliations:** ^1^ Department of General Surgery, Volgograd State Medical University, Volgograd, Russia; ^2^ Research Institute of Clinical Surgery, Pirogov Russian National Research Medical University, Moscow, Russia

**Keywords:** complicated diverticulitis, robotic technologies in complicated diverticulitis, robotic surgery, robotic urgent surgery, diverticular disease

## Abstract

**Introduction:** Complicated diverticulitis is a common abdominal emergency that often requires a surgical intervention. The systematic review and meta-analysis below compare the benefits and harms of robotic vs. laparoscopic surgery in patients with complicated colonic diverticular disease.

**Methods:** The following databases were searched before 1 March 2023: Cochrane Library, PubMed, Embase, CINAHL, and ClinicalTrials.gov. The internal validity of the selected non-randomized studies was assessed using the ROBINS-I tool. The meta-analysis and trial sequential analysis were performed using RevMan 5.4 (Cochrane Collaboration, London, United Kingdom) and Copenhagen Trial Unit Trial Sequential Analysis (TSA) software (Copenhagen Trial Unit, Center for Clinical Intervention Research, Rigshospitalet, Copenhagen, Denmark), respectively.

**Results:** We found no relevant randomized controlled trials in the searched databases. Therefore, we analyzed 5 non-randomized studies with satisfactory internal validity and similar designs comprising a total of 442 patients (184 (41.6%) robotic and 258 (58.4%) laparoscopic interventions). The analysis revealed that robotic surgery for complicated diverticulitis (CD) took longer than laparoscopy (MD = 42 min; 95% CI: [-16, 101]). No statistically significant differences were detected between the groups regarding intraoperative blood loss (MD = −9 mL; 95% CI: [–26, 8]) and the rate of conversion to open surgery (2.17% or 4/184 for robotic surgery vs. 6.59% or 17/258 for laparoscopy; RR = 0.63; 95% CI: [0.10, 4.00]). The type of surgery did not affect the length of in-hospital stay (MD = 0.18; 95% CI: [–0.60, 0.97]) or the rate of postoperative complications (14.1% or 26/184 for robotic surgery vs. 19.8% or 51/258 for laparoscopy; RR = 0.81; 95% CI: [0.52, 1.26]). No deaths were reported in either group.

**Discussion:** The meta-analysis suggests that robotic surgery is an appropriate option for managing complicated diverticulitis. It is associated with a trend toward a lower rate of conversion to open surgery and fewer postoperative complications; however, this trend does not reach the level of statistical significance. Since no high quality RCTs were available, this meta-analysis isnot able to provide reliable conclusion, but only a remarkable lack of proper evidence supporting robotic technology. The need for further evidence-based trials is important.

## Introduction

Complicated diverticulitis (CD) is a common abdominal emergency that requires surgical management whenever conservative treatment is ineffective or inappropriate. Despite decades of scientific research and continuous refinement of treatment approaches, the rates of mortality from CD are still high: 5.1% and 14.5% at 30 days and 1 year, respectively (Shaban et al., 2018). Moreover, the incidence of complicated diverticulitis among young patients has increased dramatically in recent years (Weizman and Nguyen, 2011; Shaban et al., 2018; Hunt et al., 2021).

So far, there has been no definitive consensus regarding the management of CD (Shaban et al., 2018; Sartelli et al., 2020). Most contemporary guidelines recommend surgery over conservative therapy for patients with CD because non-surgical treatment often has unsatisfactory outcomes; however, the radicality of surgery remains a matter of debate (Shah and Cifu, 2017; Swanson and Strate, 2018). Complicated Hinchey I or II diverticulitis is preferably managed with either antibiotic therapy or minimally invasive US-guided puncture, depending on the abscess size and location. By contrast, aggressive resection of the affected colonic segment with primary anastomosis should be used in patients with Hinchey III or IV diverticulitis, recurrent episodes of diverticular disease or colonic fistulas (Francis et al., 2019; Sartelli et al., 2020).

The technological aspects of surgery for CD are worth a separate discussion. Although robotic systems have earned a place in elective abdominal surgery and urology, they work best for narrow pelvic spaces and may not have an advantage over conventional procedures when used for other anatomical sites (Feinberg et al., 2016; Renshaw et al., 2018). Considering the torrential increase in robot-assisted surgical interventions and their rapid adoption in various surgical specialties, it is important to evaluate their effectiveness and safety for patients with CD.

**The aim of this study** was to compare the benefits and harms of robotic versus laparoscopic surgery in patients with complicated diverticular disease of the colon by conducting a systematic review and meta-analysis.

## Materials and methods

This section was informed by Cochrane guidelines. At the planning stage, we expected that our systematic review would include randomized controlled clinical trials (RCTs). However, no eligible RCTs were found during the initial search, so we had to consider non-randomized controlled studies for inclusion. According to the Cochrane Handbook for Systematic Reviews of Interventions, such expansion of inclusion criteria is acceptable (Chandler et al., 2021).

Inclusion criteria of participants were:

- Age above 18 years
- Involvement of any colonic segment
- Acute complicated diverticulitis (Hinchey I-IV) with long-term complications of diverticular disease (internal and external fistulas, strictures)

A study was considered for inclusion if it compared the outcomes of robot-assisted resection of the colon or its segment (with or without primary anastomosis and with or without protective loop ileostomy) to the outcomes of laparoscopic resection of the colon or its segment (with or without primary anastomosis and with or without protective loop ileostomy).

To be considered eligible for inclusion, a study was required to provide details on the following outcomes:

- Operative time
- Intraoperative blood loss
- Rate of conversion to open surgery
- Complication rate
- Length of in-hospital stay

The following databases were searched before 1 March 2023: Cochrane Central Register of Controlled Trials (CENTRAL), PubMed, Embase, CINAHL and ClinicalTrials.gov. Studies were considered eligible for inclusion if they compared the outcomes of laparoscopic vs. robotic surgery in patients with CD. A broad-search strategy was employed. The search was conducted using MeSH terms (name of the pathology and types of surgery) in the English language and Boolean operators AND and OR:

[(colon AND diverticular disease OR diverticulitis) AND (complicated OR perforated OR peritonitis) AND (robotic surgery OR robot-assisted surgery OR laparoscopy)].

The studies and systematic reviews that met the eligibility criteria were further manually searched for references to additional potentially relevant publications using the “snowball” method and citation searching. We also searched the contents of scientific journals specializing in robotic surgery.

Independently of each other, two authors (SP and TN) identified, screened and reviewed the abstracts returned by the search; then, the full-text articles were assessed for eligibility.

The following exclusion criteria were applied:

- Studies that compared robotic and laparoscopic colon surgeries to open surgical procedures without reporting the outcomes of each surgery type;
- Studies that included patients with colon tumors;
- Studies that compared open and minimally invasive surgeries.

Any disagreements were resolved through discussion with the co-authors of this publication. The study selection process was summarized in a PRISMA flow diagram (Moher et al., 2009).

The following information was retrieved from each study selected by SP and TN: study design, number of patients in each group, post- and intraoperative complications, mortality, operative time, the rate of conversion to open surgery, blood loss, and length of in-hospital stay. The accuracy of the extracted data was validated by the co-authors of this publication.

Since all the publications included in our systematic review were non-randomized controlled trials, we evaluated their internal validity using the ROBINS-I (Risk Of Bias in Non-randomized Studies - of Interventions) tool (Sterne et al., 2016). The risk of bias was assessed by two authors (SP and TN) independent of each other.

The mean difference (MD) and the risk ratio (RR) were used to measure continuous and dichotomous outcomes, respectively. The significance of differences was determined based on the 95% confidence interval (CI) and the *р*-value. We used standard formulas to estimate the mean and standard deviations from the median and range (Hozo et al., 2005). Considering the initially high level of heterogeneity, the random effects model was used for all comparisons.

The evidence included in the meta-analysis was pooled from primary controlled non-randomized studies, which is acceptable according to Chapter 24 of the Cochrane Handbook for Systematic Reviews of Interventions (Chandler et al., 2021). The data extracted from the studies that had different designs were not summarized.

Information about robotic and laparoscopic interventions contained in the selected studies was sufficient for comparative analysis. There was no need to contact their authors for further information.

We assumed that the risk of heterogeneity in non-randomized controlled studies was *a priori* high (*p* < 0.10 in the chi-squared test; I^2^ ≥ 40%) (Reeves and Wells, 2013; Chandler et al., 2021). However, since the risk of systematic bias in the primary studies was not critical, a meta-analysis was deemed possible.

Due to the small number of studies included in the analysis (*n* = 5), we did not construct a funnel plot to estimate reporting bias. The publication sources we used were represented by a variety of different databases, so we were able to avoid duplication bias.

Statistical analysis was conducted and meta-analysis graphs were constructed in RevMan 5.4. The inverse variance method was applied, as recommended by the Cochrane Handbook for Systematic Reviews of Interventions in Chapter 24 (Reeves and Wells, 2013; Chandler et al., 2021).

Statistical computations were performed by SP. Their accuracy was validated by TN.

Considering the small number of observations, no subgroup analysis was conducted.

Considering the amount and quality of evidence, sensitivity analysis was performed using random-effects and fixed-effects meta-analytic models for binary outcomes in the event of low heterogeneity (*p* > 0.10 in the chi-squared test; I^2^<40%).

To assess the risks of random errors due to sparse data, we estimated the required information size using Copenhagen Trial Unit’s Trial Sequential Analysis (TSA) software. For binary outcomes, we calculated the diversity-adjusted required information size (DARIS) based on the relative risk reduction of 10%, the control event rate obtained from the meta-analysis, α = 0.05, β = 0.20. For continuous outcomes, we calculated DARIS based on the variance estimated from the meta-analysis, the empirical mean difference, α = 0.05 and β = 0.20.

## Results

Initially, we planned to conduct a systematic review of RCTs investigating the benefits and harms of robotic surgery vs. laparoscopy in patients with complicated diverticular disease of the colon in an emergency setting. But due to the absence of relevant completed and published RCTs in the searched databases, we had to rely on the best available evidence from non-randomized studies that could be potentially aggregated into a meta-analysis. Patients undergoing elective robotic or laparoscopic surgery for CD were also included in the review.

Importantly, data generated by non-randomized clinical studies can be used in Cochrane reviews for qualitative and quantitative evidence synthesis despite a higher risk of systematic bias (Chandler et al., 2021).

**FIGURE 1:**
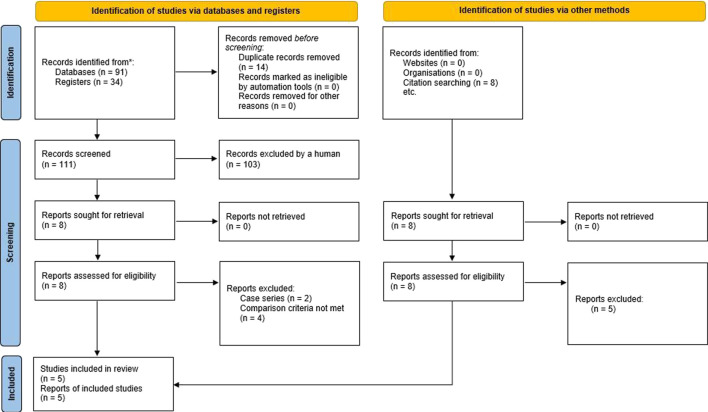
Stages of the literature search for the systematic review.

The PRISMA flow diagram showing the stages of the literature search process is provided in Figure 1.

From all the studies returned by the initial search, we identified and reviewed 16 non-randomized clinical studies investigating the use of robotic surgery in patients with CD (Maciel et al., 2014, DeLeon et al., 2014, Elliott et al., 2015, Cassini et al., 2019, Beltzer et al., 2019, Xia et al., 2019, Grass F et al., 2019, Raskin et al., 2019, Ogilvie Jr et al., 2019, Al-Temimi et al., 2020, Bastawrous et al., 2020, Bilgin. et al., 2020, Vorontsov et al., 2020, Lai et al., 2021, Abdel Jalil et al., 2021, Widder et al., 2022) (DeLeon et al., 2014; Maciel et al., 2014; Elliott et al., 2015; Al-Temimi et al., 2019; Beltzer et al., 2019; Cassini et al., 2019; Grass et al., 2019; Ogilvie et al., 2019; Raskin et al., 2019; Xia et al., 2019; Bastawrous et al., 2020; Bilgin et al., 2020; Vorontsov et al., 2020; Abdel Jalil et al., 2021; Lai et al., 2021; Widder et al., 2022). However, only 5 of these studies (Maciel et al., 2014; Elliott et al., 2015; Cassini et al., 2019; Ogilvie Jr et al., 2019; Bilginet al., 2020) were selected for further analysis. Two publications (DeLeon et al., 2014; Beltzer et al., 2019) were a case series without a control group, one publication (Raskin et al., 2019) did not describe the outcomes separately for complicated and non-complicated diverticulitis, and 3 publications (Xia et al., 2019, Grass et al., 2019, Lai et al., 2021) compared the outcomes of robotic surgery between patients with complicated and uncomplicated diverticulitis but provided no comparison between robotic surgery and laparoscopy. Two other papers (Al-Temimi et al., 2020 and Bastawrous et al., 2020) used national databases as the source of information, so their inclusion could have resulted in the incorrect interpretation of the data summarized in the meta-analysis.

Abdel Jalil et al., 2021 analyzed the outcomes of robotic vs. laparoscopic surgery in patients with either complicated diverticulitis or cancer, but the results were not reported separately for diverticulitis. Vorontsov et al. (2020) compared robot-assisted and laparoscopic techniques in patients with or without perioperative intestinal decontamination, but the results were not reported separately for each type of intervention. Widder et al., 2022 compared the outcomes of laparoscopy and robotic surgery in patients with either cancer, diverticulitis or endometriosis, but they were not reported separately for diverticulitis.

General information about the selected studies that compared the effects of robotic and laparoscopic procedures for colonic CD is provided in Table 1. There were 2 groups of patients (*n* = 442): a robotic surgery group (*n* = 184, 41.6%) and a laparoscopic surgery group (*n* = 258, 58.4%).

**TABLE 1:** General information about the primary studies included in the systematic review.

Publication (year)	Study design	Country	Surgery		
			Robotic (n)	Laparoscopic (n)	Total (n)
Maciel et al. (2014)	Non-randomized controlled	United States	*n* = 20	*n* = 55	*n* = 75
Elliott et al. (2015)	Non-randomized controlled	United States	*n* = 11	*n* = 20	*n* = 31
Cassini et al. (2019)	Non-randomized controlled	Italy	*n* = 64	*n* = 92	*n* = 156
Ogilvie Jr et al. (2019)	Non-randomized controlled	United States	*n* = 69	*n* = 69	*n* = 138
Bilgin et al. (2020)	Non-randomized controlled	Turkey	*n* = 20	*n* = 22	*n* = 42

Patient demographics are described in Table 2. Four of the reviewed studies provided information on long-term complications of diverticular disease (internal and external fistulas, strictures), another study (Cassini D et al., 2014) reported on both acute (perforation, bleeding) and long-term complications.

**TABLE 2:** Patient demographics, complications and the type of intervention used.

Publication (year)	Robotic surgery			Laparoscopic surgery		
	Men/women	Age	Complications	Men/women	Age	Complications
Maciel et al. (2014)	M-12, W-8	60.25 ± 18.75	Internal fistulas	M-27, W-28	64.35 ± 12.16	Internal fistulas
Elliott et al. (2015)	M-5, W-6	63 (44–86)	Internal and external fistulas	M-8, W-12	56 (36–79)	Internal and external fistulas
Cassini et al. (2019)	M-17, W-47	68.68 ± 11.8	Various complications	M-33, W-59	67.42 ± 13.1	Various complications
Ogilvie Jr et al. (2019)	M-25, W-44	56.9 ± 12.3	Various complications	M-28, W-41	57.9 ± 12.6	Various complications
Bilgin et al. (2020)	M-11, W-9	55.25 ± 12.4	Various complications	M-12, W-10	56.1 ± 11.6	Various complications

The internal validity of the selected primary studies was assessed using the ROBINS-I tool (Table 3); 7 domains of bias were assessed covering all parameters of perioperative comparison.

**TABLE 3:** Risk of bias assessment with ROBINS-I.

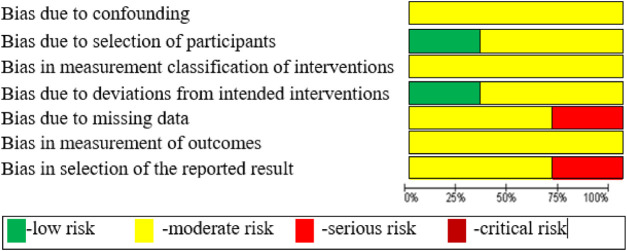

The similarity between the designs of the selected primary studies was satisfactory. The number of primary trials met the required minimum (at least two trials are needed to conduct a meta-analysis). In addition, the risk of bias was moderate and the level of internal validity was sufficient for all the selected non-randomized studies, according to the GRADE approach.

### Procedure duration

**FIGURE 2:**
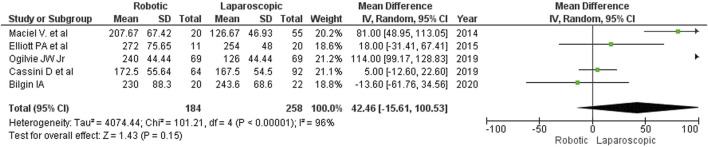
Operative time for robotic and laparoscopic interventions.

As shown in Figure 2, robotic surgery took longer than laparoscopy (MD: 42 min; 95% CI: [-16, 101]). Between the groups, the differences in operative time ranged from 5 min (Cassini D et al., 2020) to 114 min (Ogilvie J et al., 2019). Only one author (Bilgin I et al., 2020) reported that robot-assisted surgery was 14 min shorter than laparoscopy. The detected differences were, however, statistically insignificant. The authors did not specify how much time was required for docking.

**FIGURE 3:**
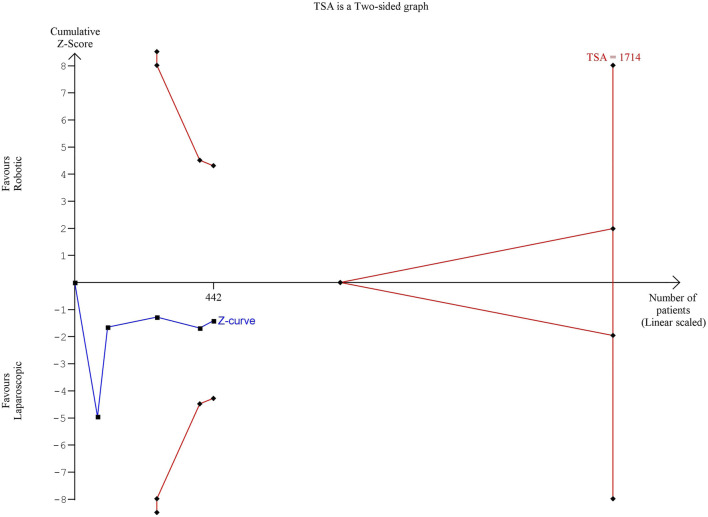
Trial Sequential analysis of operating time.

The estimated diversity-adjusted required information size (DARIS) was 1,714 participants, based on the empirical difference of 42 min, the variance (VAR) of 2,999.61, α = 5%, β = 20%, and D^2^(diversity) = 97%. The total number of patients accrued in 5 selected studies was 442, which made up 25.8% of the required information size, meaning that more trials are needed for further meta-analysis (Figure 3).

### Intraoperative blood loss

**FIGURE 4:**
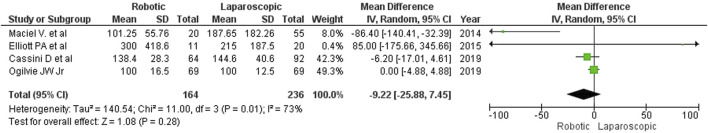
Intraoperative blood loss during robotic and laparoscopic interventions.

The volume of blood loss was reported in 4 papers, but the applied measurement techniques were not specified, except for the article by Cassini D et al. (2020), who estimated the amount of lost blood from the aspirate. Cassini D et al. and Elliott PA et al. mentioned the volume of blood transfusion, which did not differ between the groups. Robotic interventions were characterized by less intraoperative blood loss (MD: 9 mL, 95% CI: [–26, 8]), but the difference was insignificant (Figure 4).

**FIGURE 5:**
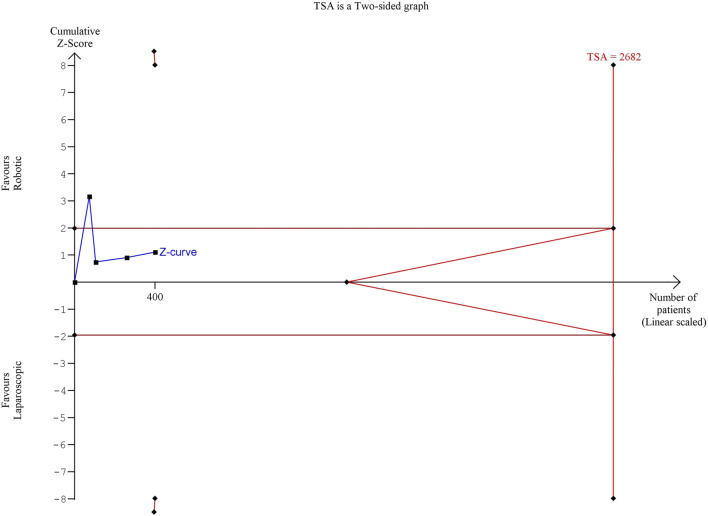
Trial Sequential analysis of intraoperative blood loss.

The estimated diversity-adjusted required information size (DARIS) was 2,682 participants, based on the empirical difference of 9 mL, VAR = 512.13, α = 5%, β = 20% and D^2^ = 93%. The total number of patients accrued in 4 selected studies was 400, constituting 14.9% of the required information size (Figure 5).

### Conversion to open surgery

**FIGURE 6:**
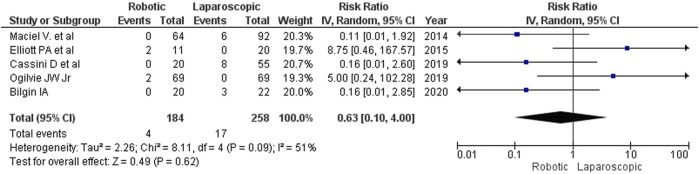
The rate of conversion to open surgery for robotic and laparoscopic interventions.

We looked at 2 types of conversion: conversion from conventional laparoscopy to open surgery and conversion from the initially robotic procedure to laparotomy. Most authors do not consider conversion an intraoperative complication, but due to its impact on the quality and duration of the postoperative period, it is important to understand the reasons for conversion. The studies included in our analysis provided these reasons. The rate of conversion to open surgery was lower for robotic interventions (2.17%, 4 of 184) than for laparoscopy (6.59%, 17 of 258), but the trend did not reach the level of statistical significance (RR 0.63, 95% CI: [0.10, 4.00]; Figure 6). The main reasons for conversion to open surgery were marked adhesions, short mesentery and high BMI. A correlational multivariate analysis of reasons for conversion was not performed in any of the analyzed studies.

**FIGURE 7:**
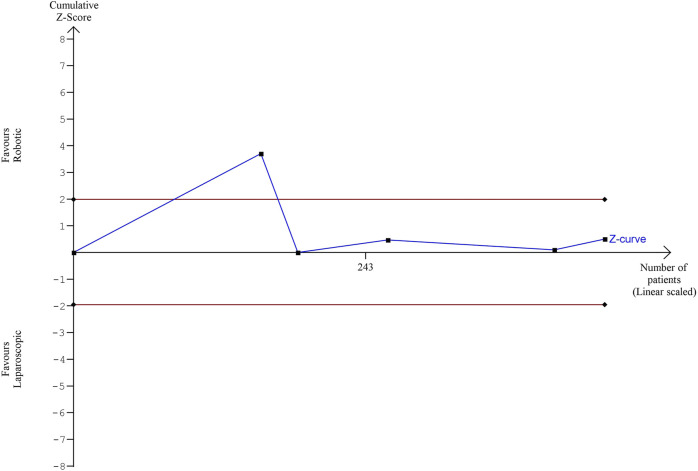
Trial Sequential analysis of conversion to open surgery.

The estimated diversity-adjusted required information size (DARIS) was 86,264 participants, based on the proportion of participants in the control group with the outcome of 6.59%, for the relative risk reduction of 10%, α = 5%, β = 20% and D^2^ = 51%. The total number of patients accrued in 5 selected studies was 442, constituting only 0.51% of the required information size. The trial sequential analysis does not show the required information size and trial sequential monitoring boundaries (Figure 7).

### Intraoperative complications

Elliott PA et al. (2015) reported no intraoperative complications for robotic and laparoscopic surgeries. Maciel V et al. (2014) mentioned 2 patients in the laparoscopy group who required intraoperative blood transfusion, but did not specify the cause of bleeding. Three other authors (Cassini D et al., 2019; Ogilvie JW Jr et al., 2019; Bilgin IA et al., 2020) did not provide sufficient data on the intraoperative complications, which made statistical analysis impossible.

### Postoperative complications

**FIGURE 8:**
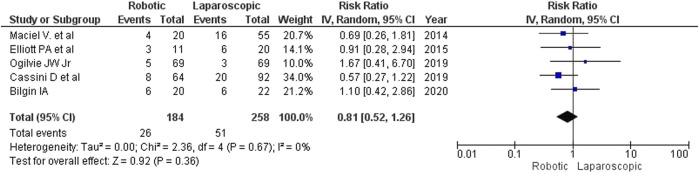
The rate of postoperative complications after robotic and laparoscopic interventions.

Differences in the rate of postoperative complications were insignificant between the intervention groups for both fixed- and random-effects models (RR 0.81, 95% CI: [0.52−1.26]), although there were fewer complications after robotic surgery (14.1%, 26/184) than after laparoscopy (19.8%, 51/258) (Figure 8). Maciel V et al. (2014) reported 2 recurrences of external colonic fistula (one in each group). Most of the reported complications were surgical site infections, pneumonia, thromboembolic complications, anastomosis leaks and postoperative ileus. The correlation analysis of factors of adverse outcomes was not performed in any of the reviewed studies.

**FIGURE 9:**
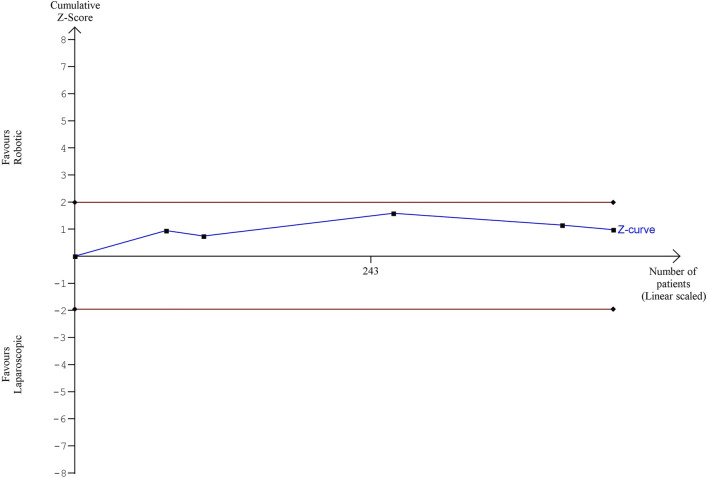
Trial Sequential analysis of postoperative complications.

The estimated diversity-adjusted required information size (DARIS) was 12,231 participants, based on the proportion of participants in the control group with the outcome of 19.8%, for the relative risk reduction of 10%, α = 5%, β = 20% and D^2^ = 0%. The total number of patients accrued in 5 selected studies was 442, constituting only 3.61% of the required information size. The trial sequential analysis does not show the required information size and trial sequential monitoring boundaries (Figure 9).

### Mortality

No deaths were reported in the early postoperative period after either type of surgery.

### Length of in-hospital stay

**FIGURE 10:**
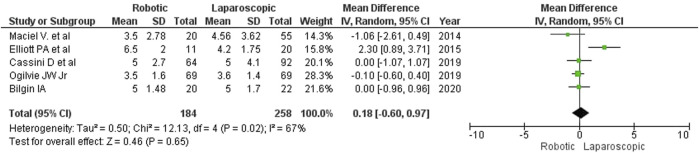
Length of hospital stay after robotic and laparoscopic surgeries.

Although laparoscopy resulted in a significantly shorter hospitalization in one of the reviewed studies (Elliott PA et al., 2015), we found no differences in the length of in-hospital stay between the groups (MD = 0.18; 95% CI: [–0.60, 0.97]; Figure 10). In Elliott’s study, the in-hospital stay was the longest (patients from the robotic surgery group had spent an average of 6.5 days in the hospital). According to Maciel V et al. (2014), the average length of in-hospital stay for patients undergoing colon resection was as short as 3.5 days.

**FIGURE 11:**
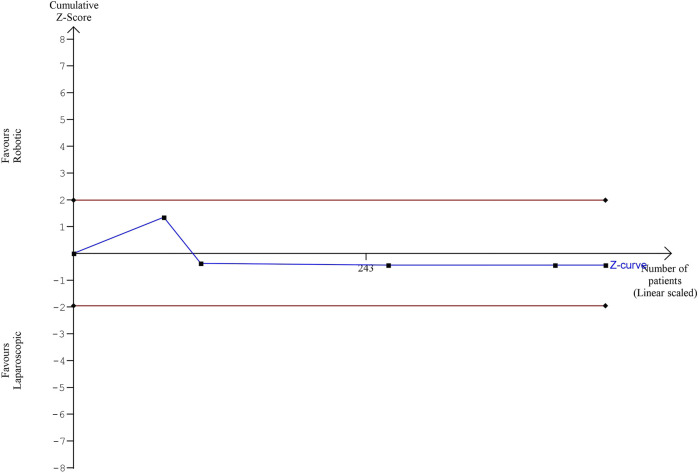
Trial Sequential analysis of hospital stay.

The estimated diversity-adjusted required information size (DARIS) was 16,629 participants, based on the empirical difference of 0.18, VAR = 4.19, α = 5%, β = 20% and D^2^ = 76%. The total number of patients accrued in 5 selected studies was 442, making up only 2.56% of the required information size (Figure 11).

Information about medical and surgical follow up was heterogeneous thus cannot be pooled. No data was provided on patient follow-up beyond 30 days by Cassini et al. (2014) Ogilvie et al. (2019) and Bilgin et al., 2020. Mean follow-up in study by Maciel V et al. (2014) was 266 days and two recurrences of a colocutaneous fistula were noted (one after robotic and one after laparoscopic surgery). Mean follow-up was 6 months in robotic and 43 months in laparoscopic groups in the study conducted by Elliott PA et al. (2015) with no fistula recurrence. One patient after laparoscopic intervention required reoperation for a trocar site hernia with small bowel obstruction.

## Discussion

Complicated diverticulitis is a significant medical and social problem. Sedentary lifestyles and unhealthy diet are likely to further increase the incidence of the disease in the future. Research shows that CD affects mostly women over 65 years of age and is associated with a high mortality rate, especially in comorbid patients. Unfortunately, the incidence of CD among younger patients has been growing steadily in the past few years (Weizman and Nguyen, 2011; Shah and Cifu, 2017).

The optimal management strategy for perforated diverticulitis is yet to be proposed. For decades, the Hartmann procedure was the procedure of choice for patients with diverticulitis complicated by peritonitis. Traditionally, it was associated with a low risk of early postoperative complications and a zero risk of anastomotic leaks. Yet the rate of stoma reversal after the Hartmann procedure was always below 50%, and complications associated with the reconstruction procedure were never included in the total statistics of complications (Shaban et al., 2018). An ongoing study *Goodbye*, *Hartmann* conducted by the World Society of Emergency Surgeons (WSES) is aimed at revisiting the two-step treatment strategy for diverticulitis. A wealth of data has been accumulated advocating a one-stage approach to the treatment of CD, involving primary anastomosis. In addition to perforated diverticulitis and colonic fistulas, for which colonic resection has no alternative, indications for the radical treatment of CD are now expanding to include mild forms of inflammation (Hinchey 1 and 2) and a previous history of single flare-ups (Shah and Cifu, 2017; Shaban et al., 2018; Francis et al., 2019; Sartelli et al., 2020).

Robotic surgery is being increasingly used on patients with abdominal emergencies (Felli et al., 2014; Kubat et al., 2016; Cubas et al., 2021). However, its outcomes are mostly presented in the literature as clinical cases. For example, Cubas et al., 2021 performed robotic diaphragm repair for incarcerated Morgagni hernia, and Felli et al., 2014 performed a right-sided hemicolectomy on a patient with a bleeding tumor. In addition to complex anatomical areas and extensive interventions, where robotic technology is legitimately expected to be effective, research studies look at the use of surgical robots in standard and technically simple procedures, such as single-port cholecystectomy. Coloproctology is an example of a surgical field that has harnessed the advances in robotic technology (Felli et al., 2014; Cassini et al., 2019; Anderson et al., 2020). Still, there is a paucity of studies investigating the effects of robotic surgery in patients with CD.

There are a few obstacles to the wider spread of robot-assisted technology in clinical practice. First, robotic surgery platforms and consumables are quite costly. Second, elective surgeons may face certain difficulties when having to perform a robotic procedure in the emergency setting. Cassini D et al. (2019) compared the level of stress experienced by the surgeons performing robotic and laparoscopic procedures for CD. The level of stress and the intensity of effort were evaluated using a Cassini-Grieco-Depalma (CGD) Stress Score. According to the study, robotic technology was less labor-intensive and accompanied by a significantly lower level of stress for the surgeon in comparison with laparoscopy. However, Cassini’s sample size was small (6 surgeons), raising the need for further research (Cassini et al., 2019).

The overall risk of bias was moderate in the selected non-randomized studies, which allowed us to summarize their results in a meta-analysis. No convincing differences were detected in the outcomes of robotic vs. laparoscopic surgery in patients with CD. Although robotic surgery usually took longer than laparoscopy, it was associated with less blood loss and had a lower rate of conversions and complications. This trend, however, did not reach the level of statistical significance due to the small number of patient groups and studies.

The results of our statistical analysis should be interpreted with caution because the analyzed data obtained from non-randomized studies cannot ensure a high level of protection against systematic bias. Besides, the meta-analysis covered only 5 studies, so its statistical power may be insufficient. That said, we used a random-effects model, which prevents incorrect conclusions due to very high heterogeneity and small sample sizes.

The most statistically powerful study of surgical interventions for complicated and uncomplicated diverticular disease was conducted by Raskin ER et al. in 2019. The study analyzed the outcomes of 12,652 patients undergoing sigmoidectomy (robotic surgery: 10%; laparoscopy: 61%; open surgery: 29%) (Raskin et al., 2019). After adjusting for confounding factors with Propensity Score-Matching and establishing a sample size of over 1,000 observations per group, the authors discovered that robotic surgery was associated with a shorter in-hospital stay and a lower rate of postoperative complications than laparoscopy and open surgery. The rate of intraoperative complications did not differ between the groups. The rate of conversion to open surgery was lower for robotic surgery than for laparoscopy but robotic surgery was more time-consuming than laparoscopy or open surgery. Unfortunately, Raskin et al. (2019) did not compare the outcomes of robotic and laparoscopic interventions in the subgroup of patients with CD; therefore, we could not use its results in the meta-analysis.

There are 3 more published meta-analyses that compare the outcomes of robotic vs. laparoscopic surgery in patients with diverticulitis (Giuliani et al., 2021; Solaini et al., 2022; Larkins et al., 2022), but they do not focus on complicated diverticulitis specifically (Giuliani et al., 2021; Larkins et al., 2022; Solaini et al., 2022). Thus, Larkins (2022) compared robotic and laparoscopic procedures in patients with diverticular disease but the patients were not stratified based on the type of complications of the disease. Giuliani et al. (2021) compared the outcomes of robotic and laparoscopic interventions in patients with left-sided colonic diverticular disease, and Solaini et al. (2022) studied the outcomes of left colectomy in patients with diverticulitis and neoplasms. It should also be noted that the aforementioned authors (Giuliani et al., 2021; Larkins et al., 2022; Solaini et al., 2022) did not conduct Trial Sequential Analysis in their studies.

Similar to the publications by Giuliani G. et al., Solaini L et al. and Larkins K, our systematic review summarizes and broadens the existing evidence on the outcomes of robotic vs. laparoscopic surgery in patients with colonic diverticular disease, providing a rationale for further research.

Just like us, the authors of the publications failed to find any relevant RCTs, but unlike us their analysis did include studies with not only prospective but also retrospective data. We did not do so because non-randomized trials with different study design features are susceptible to different biases (Sterne et al., 2016; Chandler et al., 2021).

This review analyzes and summarizes the results of the few non-randomized studies in which potential risk for bias is greater than in RCT’s. In the presence of confounding bias the results should be interpreted with caution. The personal preferences of surgeons and experience in robotic surgery can be mentioned as possible predisposing factors for confounders. Moreover, already published meta-analisys (Larkins K et al., 2022) considers the importance of the initial stage of the learning curve, in which surgeons select less complex cases for robotic operations as a confounder (Larkins et al., 2022).

It should also be noted that we report no data about any economic aspects as they were beyond the scope of our current research. The cost analysis is a determining factor in the evaluation of any new technology. A detailed systematic analysis of 30 papers on the cost-effectiveness of robotic operations noted significant heterogeneity in research with respect to design and methodologies for calculating cost-effectiveness. There were no RCTs among these works, most of the studies were single-center. The majority of studies legitimately noted the higher cost of robotic operations compared to laparoscopic and open procedures. The average incremental cost of robotic surgery was $4,625 per patient compared to laparoscopic surgery (Ho et al., 2011). These data were subsequently confirmed by Khorgami Z who performed an analysis of the added costs of robotic-assisted versus laparoscopic surgery based on an evaluation of 91,630 operations. The incremental cost compared to laparoscopic surgery was 6%–25% depending on the type of surgery (Khorgami et al., 2019). Additionally, Solaini et al., 2022 have failed to pool the results of published trials which compared cost values of robotic and laparoscopic techniques (Solaini et al., 2022).

Since economic analysis is a topic of a separate work and the authors of included manuscripts did not provide data on the cost of operations, we were unable to include these data in our analysis. We consider cost–minimization analysis (CMA) and cost–utility analysis (CUA) for further targeted research.

Overviewing the mentioned above limitations its necessary to point out that contributing to evidence-based practice presented meta-analysis definitely has an advantage over “empty” reviews that merely state the absence of eligible RCTs and the inability of establishing differences between compared groups at the first level of evidence.

## Conclusion

Similar to laparoscopy, robotic surgery can be used in clinical practice to manage complicated diverticular disease of the colon**.** According to the meta-analysis presented in this article, robotic surgery is a reliable technique for managing complicated diverticulitis. It is associated with a trend toward a lower rate of conversion to open surgery and fewer postoperative complications; however, this trend does not reach the level of statistical significance. Since no RCTs were available, this meta-analysis is unable to provide reliable conclusions, but only a remarkable lack of proper evidence supporting robotic technology. The need for further evidence-based trials is important.

## Data Availability

The original contributions presented in the study are included in the article/Supplementary material, further inquiries can be directed to the corresponding author.
